# Synthesis, Identification, Computer-Aided Docking Studies, and ADMET Prediction of Novel Benzimidazo-1,2,3-triazole Based Molecules as Potential Antimicrobial Agents

**DOI:** 10.3390/molecules26237119

**Published:** 2021-11-25

**Authors:** Huda R. M. Rashdan, Aboubakr H. Abdelmonsef, Mortaga M. Abou-Krisha, Tarek A. Yousef

**Affiliations:** 1 Chemistry of Natural and Microbial Products Department, Pharmaceutical and Drug Industries Research Institute, National Research Institute, Dokki, Cairo 12622, Egypt; 2 Chemistry Department, Faculty of Science, South Valley University, Qena 83523, Egypt; aboubakr.ahmed@sci.svu.edu.eg (A.H.A.); mmaboukrisha@imamu.edu.sa (M.M.A.-K.); 3 Chemistry Department, College of Science, Imam Mohammad Ibn Saud Islamic University (IMSIU), Riyadh 11623, Saudi Arabia; 4 Mansoura Laboratory, Department of Toxic and Narcotic Drug, Forensic Medicine, Medicolegal Organization, Ministry of Justice, Mansoura 35511, Egypt

**Keywords:** benzimidazole, 1,3,4-thiadiazole, 1,2,3-triazoles, antimicrobial activity, docking study

## Abstract

2-azido-1*H*-benzo[d]imidazole derivatives **1a**,**b** were reacted with a β-ketoester such as acetylacetone in the presence of sodium ethoxide to obtain the desired molecules **2a**,**b**. The latter acted as a key molecule for the synthesis of new carbazone derivatives **4a**,**b** that were submitted to react with 2-oxo-*N*-phenyl-2-(phenylamino)acetohydrazonoyl chloride to obtain the target thiadiazole derivatives **6a**,**b**. The structures of the newly synthesized compounds were inferred from correct spectral and microanalytical data. Moreover, the newly prepared compounds were subjected to molecular docking studies with DNA gyrase B and exhibited binding energy that extended from −9.8 to −6.4 kcal/mol, which confirmed their excellent potency. The compounds **6a**,**b** were found to be with the minimum binding energy (−9.7 and −9.8 kcal/mol) as compared to the standard drug ciprofloxacin (−7.4 kcal/mol) against the target enzyme DNA gyrase B. In addition, the newly synthesized compounds were also examined and screened for their in vitro antimicrobial activity against pathogenic microorganisms *Staphylococcus aureus*, *E. coli,* *Pseudomonas aeruginosa*, *Aspergillus niger*, and *Candida albicans*. Among the newly synthesized molecules, significant antimicrobial activity against all the tested microorganisms was obtained for the compounds **6a**,**b**. The in silico and in vitro findings showed that compounds **6a**,**b** were the most active against bacterial strains, and could serve as potential antimicrobial agents.

## 1. Introduction

Nitrogen-containing heterocyclic analogues have received great interest in drug discovery because of their well-known activity in pharmaceutical and medicinal fields [1,2,3,4]. Benzimidazole derivatives represent an important heterocyclic class of active therapeutic agents because of their wide spectrum of biological and pharmaceutical applications including antibacterial, antifungal, antiviral, anticancer, antidiabetic, anticonvulsant, and anti-HIV agents [5,6]. In addition, 1,2,3-triazoles have attracted a great deal of interest from medicinal chemists in the design and development of potential drug candidates due to their high pharmacological properties such as antimicrobial, antitubercular, CNS depressant, and antihypertensive activities [7,8,9,10,11,12]. They have diverse applications in drug development for the treatment of some diseases including cancer, inflammation, malaria, and tuberculosis [13,14,15]. 

**Figure 1:**
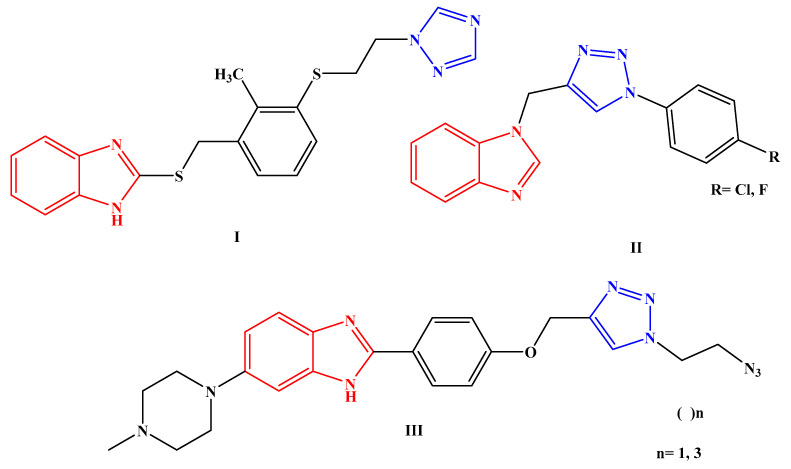
Hybrid molecules bearing benzimidazole and triazole moieties I–III as antibacterial agents.

A wide range of antibacterial properties have been gained in hybrid molecules containing benzimidazole and triazole moieties, as shown in Figure 1. 

On the other hand, DNA gyrase is considered an important bacterial enzyme that is involved in the control of topological transitions of DNA [16,17]. In addition, it is the intercellular target for a number of antibacterial agents as a paradigm for other DNA topoisomerases [18]. Therefore, DNA gyrase B has been selected as a therapeutic target for the identification and development of antimicrobial agents [19,20,21].

In light of the potential therapeutic properties of these heterocyclic compounds and with the contribution of our research work [18,22,23,24,25,26,27,28,29,30,31] to identify a new class of antimicrobial agents, a novel set of hybrid derivatives bearing benzimidazole and triazole moieties was synthesized. Their antimicrobial activities were performed in vitro. Moreover, the molecular docking study [32,33,34] was performed both to support the antibacterial activity and to understand the binding mode of the interactions of the screened compounds against the binding site of the target enzyme DNA gyrase B. Furthermore, the in silico absorption, distribution, metabolic, excretion, and toxicity (ADMET) and drug-likeness of the prepared compounds were also calculated to identify their bioavailability and toxicity.

## 2. Results and Discussion 

### 2.1. Chemistry

**Scheme 1:**
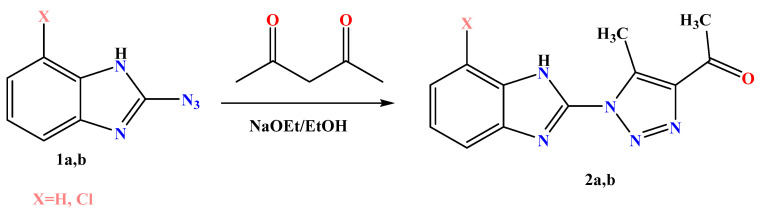
Synthesis of acetyl triazole derivatives **2a**,**b**.

In the present study, a preparation of the starting materials 1-(5-methyl-1-(7-aryl-1*H*-benzo[d]imidazol-2-yl)-1*H*-1,2,3-triazol-4-yl) ethan-1-one **2a**,**b**, were obtained by the reaction of benzo[*d*]imidazoles **1a** and/or **1b** with *β*-ketoester acetylacetone in the presence of sodium ethoxide in ethanol under reflux (Scheme 1). Their structures were confirmed using spectral data and elemental analysis. The FT-IR spectrum of **2a** and **2b** exhibited significant strong absorption bands at *v* 1715 and 1705 for the carbonyl group, respectively, indicated the formation of the acetyl group. In addition, the ^1^H-NMR spectrum of **2a** and **2b** showed characteristic singlet signals at *δ* 2.10 and 2.42 ppm for compound **2a** and at *δ* 2.16 and 2.47 ppm for compound **2b** for the two methyl groups formed in each compound. The ^13^C-NMR of **2a** exhibited two signals at *δ* 9.52 and 27.42 ppm for the two methyl groups and at 195.9 ppm for the carbonyl group. Additionally, the ^13^C-NMR of **2b** exhibited two signals at *δ* 9.58 and 27.45 ppm for the two methyl groups and at 194.82 ppm for the carbonyl group. Moreover, the structures were also supported by their mass spectrum, which agree with their molecular formula. 

**Scheme 2:**
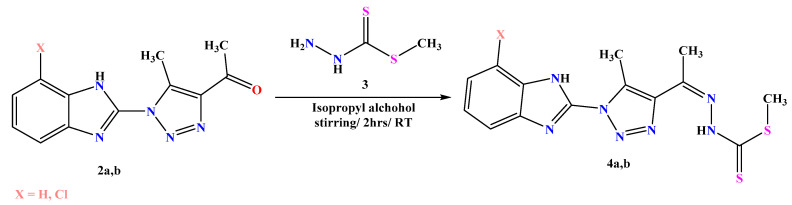
Synthesis of carbodithioate derivatives **4a**,**b**.

The two acetyl triazole derivatives were stirred with methyl hydrazinecarbodithioate **3** in isopropyl alcohol for 2h at RT to afford the desired carbodithioate derivatives **4a**,**b** (Scheme 2). The structures of **4a** and **4b** were confirmed on the basis of their spectral and microanalytical data. The FT-IR spectra of the two compounds were devoid of any signals for carbonyl groups. The ^1^H-NMR spectrum of **4a** showed three singlet signals at *δ* 2.16, 2.36, and 2.47 ppm for the three methyl groups and two singlet signals at *δ* 8.95 and 11.58 ppm for the NH groups. The ^1^H-NMR spectrum of **4b** exhibited three singlet signals at *δ* 2.18, 2.32, and 2.42 for the methyl groups and two singlet signals at *δ* 8.98 and 11.47 ppm for the NH groups. Their mass spectra agreed with their molecular formulas. 

**Scheme 3:**
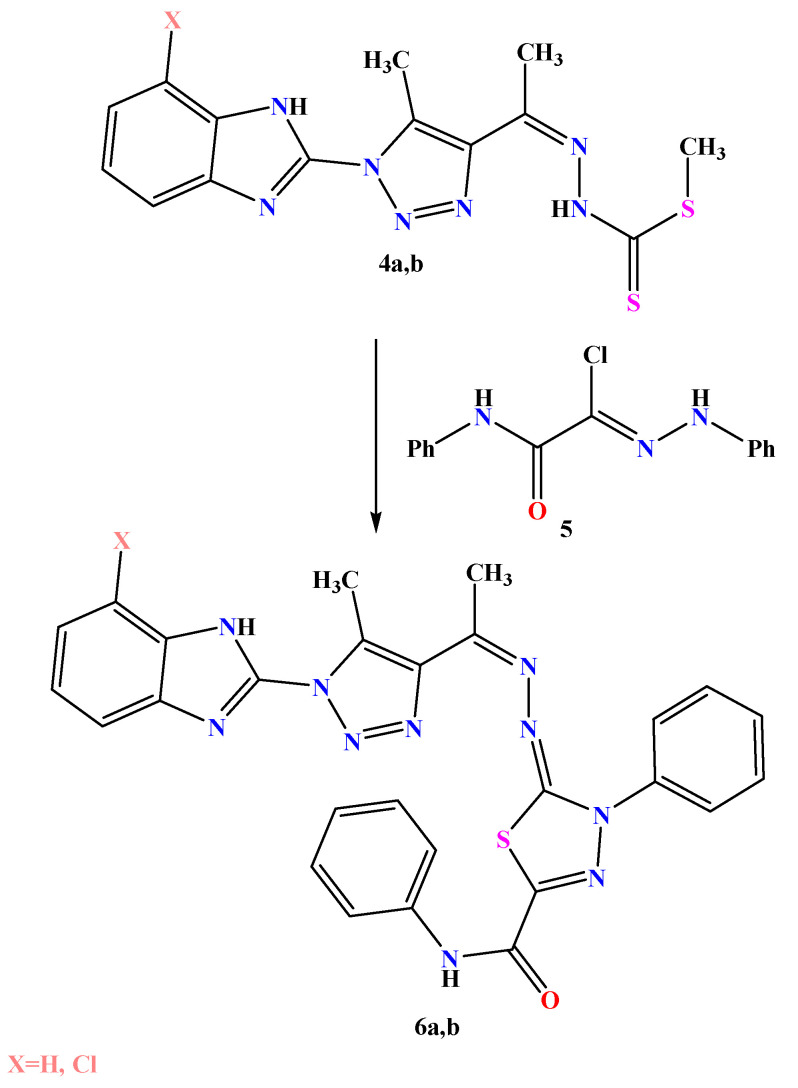
Synthesis of thiadiazole derivatives **6a**,**b**.

Compounds **4a** and **4b** were treated with 2-oxo-N-phenyl-2-(phenylamino)aceto hydrazonoyl chloride in ethanol containing a catalytic amount of TEA (2–3 drops) to obtain the corresponding 1,3,4-thiadiazole derivatives **6a**,**b** (Scheme 3). Their chemical structures were inferred from their data, while the FT-IR spectrum of compound 6a showed a strong absorption band at 1685 for the carbamide group. Additionally, compound 6b exhibited a strong absorption band at 1692 for the carbamide group. Their ^1^H-NMR and ^13^C-NMR spectra showed characteristic signals; the ^1^H-NMR spectrum of compound **6a**, for example, showed significant two signals at *δ* 2.38 and 2.46 ppm for the two methyl groups in addition to the two singlet signals at *δ* 9.81 and 11.52 ppm for the two NH groups. Its ^13^C-NMR spectrum showed two significant signals at 10.1 and 20.5 ppm for the two methyl groups. Their mass spectra supported their molecular formula. 

### 2.2. Computational Approach

#### 2.2.1. Molecular Docking Study 

**Figure 2:**
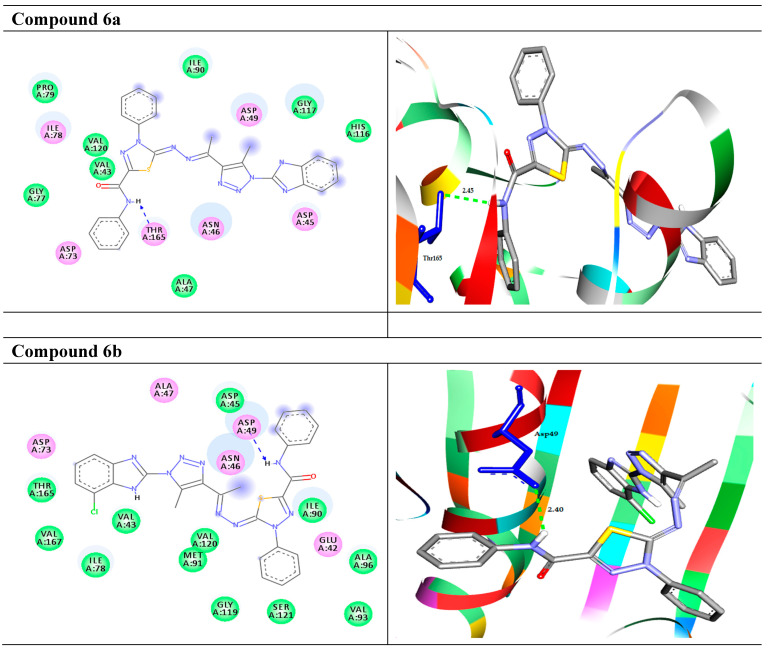
The molecular interactions of the best docked compounds **6a** and **6b** with the target enzyme DNA gyrase B. Left side (2D): the residues are represented in 3 letter codes. Hydrogen bonds are represented by green and blue lines and pi-interactions are represented by orange lines. Right side (3D): the docked compounds are represented by gray stick models, and the active site pockets are shown by blue stick models. H-bond interactions are shown in green dashed lines. π-interactions are shown in orange lines.

Based on the bacterial activity results, the compounds (**1a**–**6b**) were docked to the target enzyme DNA gyrase subunit b to check their binding profile. The docking results exhibited that the compounds (**1a**–**6b**) were fitted well in the active site pockets of the target and possessed good binding energy values ΔG (−6.4 to −9.8 kcal/mol), as summarized in Table 1 and Figure 2. It has been observed that the derivative **1a** and **1b** formed arene- σ with the target enzyme through the amino acid residue Asn46 at distances of 3.91 and 3.93 Å, respectively. Moreover, **1a** docked to the target through one HB with the residue Val71 at 1.87 Å. Compounds **2a** and **2b** showed the same interactions with the target enzyme as they formed three HB and one arene–cation interactions with the residues Ala47, Thr165, and Arg76. Compound **4a** formed two HB interactions with Thr34 at 2.98 and 3.00 Å. On the other hand, derivative **4b** exhibited one HB interaction with Asp49 at 2.20 Å. Compound **6a** showed one HB with the target through Thr164 at 2.45. Finally, compound **6b,** with the highest binding affinity of −9.8 kcal/mol, docked to the target DNA gyrase B through one HB with the residue Asp49 at 2.40 Å. The 2D and 3D representations of the intermolecular interactions of the best docked compounds **6a** and **6b** with the target enzyme are shown in Figure 2. The interactions of the other compounds and the reference drug are shown in Figure S1 in the Supplementary File Section.

On the other hand, the standard drug ciprofloxacin docked to the target enzyme with binding energy ΔG (−7.4 kcal/mol), and showed three HB interactions with the residues Arg76, Thr165, and Val43 at 3.00, 2.74, and 2.04 Å, respectively (Figure 2).

The results justified that the compounds **6a**,**b** firmly docked to the active site pockets of the target enzyme of DNA gyrase subunit B, which functionally participates in DNA inhibitions.

#### 2.2.2. Predictive ADMAT and Drug-Likeness of the Compounds 

With the help of free accessible servers such as admetSAR, mol inspiration, and SwissADME, the pharmacokinetics, physicochemical, and drug-likeness of the newly prepared compounds are summarized in Table 2. ADMET analysis of the screened compounds exhibited that they have good absorption properties (%HIA) ranging from 99.57 to 100%. For distribution, the compounds do not permeate the blood–brain barrier (BBB) except compound **1a**. Moreover, the molecules were negative in the AMES toxicity and carcinogenicity test, which suggests that they are non-mutagenic. In addition, the physiochemical properties results exhibited acceptable values as the compounds had M.W (molecular weight) (<500 g/mol), except compounds **6a**,**b**. The partition coefficients of the compounds were <5. The TPSA (topological surface areas) were found to be in the favorable range (<140). Further, the HBA (H-bond acceptors) and HBD (donors) in the compounds comply with Lipinski′s rule of five and were found to be in the acceptable range. Overall, we can conclude that they showed good absorption, distribution, and oral bioavailability within the body by not violating Lipinski′s rule of five more than once.

### 2.3. Antimicrobial Activity

All the newly synthesized molecules were screened for their antimicrobial activity against the pathogenic microorganisms *Aspergillus niger*, *candida albicans*, *Pseudomonas aeruginosa*, *Staphylococcus aureus,* and *E coli* using the agar diffusion method. The inhibition zone diameters were measured and compared with that of the standard drugs, as tabulated in Table 3. Ciprofloxacin (5µg/disc) and nystatin (100 units/disc) were used as standard antibacterial and antifungal drugs, respectively. The results of the antimicrobial screening showed that compounds **6a** and **6b** had strong activity against all the tested pathogenic microbes. In addition, compounds **2a** and **4a** only showed effects against the Gram-negative and Gram-positive bacteria and had no effect on the fungi tested. On the other hand, compound **2b** showed strong effects against the fungi tested and no effect against the bacteria tested. Finally, compound **4b** revealed moderate activity against all the microorganisms tested, except *Staphylococcus aureus.*

## 3. Experimental

### 3.1. Chemistry

#### 3.1.1. Experimental Instrumentation

All melting points were determined on an electrothermal apparatus and are uncorrected. IR spectra were recorded (KBr discs) on a Shimadzu FT-IR 8201 PC spectrophotometer. ^1^H-NMR and ^13^C-NMR spectra were recorded in (CD3)_2_SO solutions on a BRUKER500 FT-NMR system spectrometer, and chemical shifts are expressed in ppm units using TMS as an internal reference. Mass spectra were recorded on a GC-MS QP1000 EX Shimadzu. Elemental analyses were carried out at the Microanalytical Center of Cairo University.

#### 3.1.2. General Procedures for Synthesis of Derivatives **2a**,**b**

2-azido-1*H*-benzo[d]imidazole **1a** and/or 2-azido-7-chloro-1*H*-benzo[d]imidazole **1b** (10 mmol) were reacted with acetylacetone (2 mL, 20 mmol) under reflux in ethanol containing sodium ethoxide (0.5 g, 10 mmol) for 5 h. The resulting solid that formed after cooling was collected and recrystallized from ethanol to afford **2a** and **2b**, respectively.

1-(1-(1*H*-benzo[d]imidazol-2-yl)-5-methyl-1*H*-1,2,3-triazol-4-yl)ethan-1-one derivatives **2a**

White crystals, m.p. 218–220 °C, yield: 58%; FT-IR (KBr, cm^−1^): *v* 3461(NH), 2919, 2852 (CH), 1715(C=O), 1618 (C=N); 1600(C=C); ^1^H-NMR (500 MHz, DMSO-d*6*): *δ* 2.10 (s, 3H, CH_3_), 2.42 (s, 3H, CH_3_), 7.21 (d, 2H, *J* = 10Hz, ArH), 7.54 (d, 2H, *J* = 10Hz, ArH), 11.52 (s, 1H, NH); ^13^C-NMR (100 MHz, DMSO-*d_6_*): *δ* 9.5 (CH_3_), 27.4 (CH_3_), 115.2, 123.4, 133.1, 138.3, 138.7, 141.5, 195.9 (C=O); ESI-MS: *m*/*z*(%): 241 [M^+^] (11), 225 (12), 212 (13), 177 (50), 166 (18), 77 (91), 65 (100). Anal. Calcd. for C_12_H_11_N_5_O (241): C, 59.74; H, 4.60; N, 29.03% found: C, 59.78; H, 4.52; N, 29.01%.

1-(1-(7-chloro-1*H*-benzo[d]imidazol-2-yl)-5-methyl-1*H*-1,2,3-triazol-4-yl) ethan-1-one **2b**

White crystals, m.p. 228–230 °C, yield: 52%; FT-IR (KBr, cm^−1^): *v* 3450 (NH), 2921, 2858 (CH), 1705 (C=O), 1622 (C=N); 1600 (C=C); 1H-NMR (500 MHz, DMSO-d6): δ 2.16 (s, 3H, CH3), 2.47 (s, 3H, CH3), 7.21–7.25 (m, 3H, ArH), 11.58 (s, 1H, NH); 13C-NMR (100 MHz, DMSO-d6): δ 9.5 (CH3), 27.4 (CH3), 113.5, 120.2, 124.4, 133.1, 138.3, 140.7, 141.5, 194.8 (C=O); ESI-MS: *m*/*z*(%): 275 [M^+^] (15), 273 (17), 265 (18), 238 (12), 222 (14), 170 (100), 166 (28), 65 (50). Anal. Calcd. for C12H10ClN5O (275): C, 52.28; H, 3.66; N, 25.40% found: C, 52.34; H, 3.62; N, 25.37%.

#### 3.1.3. General Procedures for Synthesis of **4a**,**b**

To a solution of **2a** and/or **2b** (10 mmol) in 2-propanol (20 mL), methyl hydrazine carbodithioate **3** (1.22 g, 10 mmol) was added. The mixture was stirred at RT for 2 h. The solid product was filtered off, recrystallized from Acetic acid to afford the target molecules **4a** and **4b**, respectively.

Methyl-2-(1-(1-(1*H*-benzo[d]imidazol-2-yl)-5-methyl-1*H*-1,2,3-triazol-4-yl) ethylidene) hydrazine-1-carbodithioate **4a**

Yellow crystals, m.p. 240–242 °C, yield: 75%; FT-IR (KBr, cm^−1^): *v* 3417 (broad, NH), 1622 (C=N); 1600 (C=C); ^1^H-NMR (500 MHz, DMSO-*d_6_*): *δ* 2.16 (s, 3H, CH_3_), 2.36 (s,3H, CH_3_), 2.47 (s, 3H, CH_3_), 7.35–7.85 (m, 4H, ArH), 8.95 (s,1H, NH), 11.58 (s, 1H, NH); ^13^C-NMR (100 MHz, DMSO-*d_6_*): *δ* 10.0 (CH_3_), 16.9 (CH_3_), 18.7 (CH_3_), 115.2, 123.2, 138.7, 141.5, 145.6 (C=N), 200.0 (C=S); ESI-MS: *m*/*z*(%): 345 [M^+^] (42), 325 (18), 293 (10), 271 (8), 240 (12), 222 (14), 197 (15), 181 (20), 166 (28), 77 (14), 50 (10). Anal. Calcd. for C_14_H_15_N_7_S_2_ (345): C, 48.68; H, 4.38; N, 28.38% found: C, 48.57; H, 4.32; N, 28.31%.

Methyl-2-(1-(1-(7-chloro-1*H*-benzo[d]imidazol-2-yl)-5-methyl-1*H*-1,2,3-triazol-4-yl) ethylidene) hydrazine-1-carbodithioate **4b**

Yellow crystals, m.p. 263–265 °C, yield: 75%; FT-IR (KBr, cm^−1^): *v* 3419 (broad, NH), 1628 (C=N); 1612 (C=C); ^1^H-NMR (500 MHz, DMSO-*d_6_*): *δ* 2.18 (s, 3H, CH_3_), 2.32 (s,3H, CH_3_), 2.42 (s, 3H, CH_3_), 7.14–7.42 (m, 3H, ArH), 8.98 (s,1H, NH), 11.47 (s, 1H, NH); ^13^C-NMR (100 MHz, DMSO-*d_6_*): δ 9.1 (CH_3_), 16.9 (CH_3_), 19.7 (CH_3_), 117.2, 124.2, 139.7, 142.5, 142.6, 143.5 (C=N), 197.1 (C=S); ESI-MS: *m*/*z*(%): 379 [M^+^] (12), 352 (17), 287 (51), 252 (18), 250 (12), 227 (14), 170 (15), 165 (20), 70 (14), 65 (100). Anal. Calcd. for C_14_H_14_ClN_7_S_2_ (379): C, 44.26; H, 3.71; N, 25.81% found: C, 44.32; H, 3.65; N, 25.73%.

#### 3.1.4. General Procedures for Synthesis of Compounds **6a**,**b**

To a mixture of methyl carbodithioate **4a** and/or **4b** (1 mmol) and the appropriate 2-oxo-*N*-phenyl-2-(phenylamino) aceto hydrazonoyl chloride (0.27 gm, 1 mmol) in ethanol (20 mL), TEA (0.5 mL) was added; the mixture was stirred at RT for 2–4 h (monitored with TLC). The resulting solid was collected and recrystallized from the proper solvent to obtain the desired 1,3,4-thiadiazolines **6a** and **6b**, respectively.

5-(1-(1-(1-*H*-benzo[d]imidazol-2-yl)-5-methyl-1*H*-1,2,3-triazol-4-yl)ethylidene) hydrazono)-N,4-diphenyl-4,5-dihydro-1,3,4-thiadiazole-2-carboxamide **6a**

Yellow crystals, m.p. 251–253 °C, yield: 67%; FT-IR (KBr, cm^−1^): *v* 3435 (broad, NH), 1685 (C=O), 1600 (C=N), 1590 (C=C); ^1^H-NMR (500 MHz, DMSO-*d_6_*): *δ* 2.38 (s,3H, CH3), 2.46 (s, 3H, CH_3_), 7.32–7.91 (m, 14H, ArH), 9.81 (s,1H, NH), 11.52 (s, 1H, NH); ^13^C-NMR (100 MHz, DMSO-d6): δ 10.1 (CH_3_), 20.5 (CH_3_), 115.20, 122.40, 123.06, 129.60, 133.7, 138.30, 138.90, 141.50 (C=N), 158.20 (C=N), 160.00 (C=O), 164.60 (C=N); ESI-MS: *m*/*z*(%): 534 [M^+^] (12), 520 (16), 492 (26), 478 (14), 425 (17), 357 (17), 312 (10), 287 (51%), 252 (18), 251 (12), 236 (14), 172 (15), 167 (21), 77 (12), 50 (100). Anal. Calcd. for C_27_H_22_N_10_OS (534): C, 60.66; H, 4.15; N, 26.20% found: C, 60.72; H, 4.11; N, 26.15%.

5-((1-(1-(7-chloro-1*H*-benzo[d]imidazol-2-yl)-5-methyl-1*H*-1,2,3-triazol-4-yl) ethylidene)hydrazono)-N,4-diphenyl-4,5-dihydro-1,3,4-thiadiazole-2-carboxamide **6b**

Yellow crystals, m.p. 282–284 °C, yield: 65; FT-IR (KBr, cm^−1^): *v* 3447 (broad, NH), 1692 (C=O), 1611 (C=N), 1595 (C=C); ^1^H-NMR (500 MHz, DMSO-*d_6_*): *δ* 2.32 (s,3H, CH_3_), 2.48 (s, 3H, CH_3_), 7.37–7.81 (m, 13H, ArH), 9.82 (s,1H, NH), 11.57 (s, 1H, NH); ^13^C-NMR (100 MHz, DMSO-*d_6_*): δ 10.1 (CH_3_), 20.5 (CH_3_), 115.2, 122.4, 123.0, 129.6, 133.7, 138.3, 138.9, 148.5 (C=N), 156.2 (C=N), 162.0 (C=O), 165.6 (C=N); ESI-MS: *m*/*z*(%): 570 [M + 2] (15) 568 [M^+^] (14), 530 (12), 467 (30), 420 (16), 397 (12), 342 (18), 278 (61), 251 (11), 245 (17), 226 (14), 167 (15), 165 (22), 77 (17), 50 (100). Anal. Calcd. for C_27_H_21_ClN_10_OS (568): C, 56.99; H, 3.72; N, 24.61% found: C, 56.92; H, 3.65; N, 24.58%.

### 3.2. Docking Study 

In the present study, an in silico docking study was carried out to evaluate the binding geometries of the ligand molecules with the target enzyme. To understand the interactions of all the newly prepared compounds with DNA gyrase B, the crystallographic structure of the enzyme was downloaded from the Protein Data Bank (PDB: 1KZN) with resolution 2.3 Å [35]. In addition, the 2D structures of the ligand molecules were drawn using ChemDraw 16 (CambridgeSoft, Massachusetts, MA, USA), then converted to SDF format using Open Babel GUI [36]. The docking-based virtual screening approach was achieved using PyRx-tool [37]. To analyze the docking results, the discovery studio 3.5 was employed. In silico ADMET and drug-likeness predictions of the molecules were performed using free accessible servers such as admetSAR, mol inspiration, and SwissADME.

### 3.3. Antimicrobial Activity

#### 3.3.1. Antimicrobial Assay

The antimicrobial activities of the tested compounds were tested against the pathogenic microorganisms *Aspergillus niger* and *candida albicans* (NRRL Y-477) using sabouraud dextrose agar medium and *Pseudomonas aeruginosa* (NRRL B-23, Gram-negative bacteria), *E. coli* (*E. coli O157*), and *Staphylococcus aureus* (NRRL B-313, Gram-positive bacteria) using nutrient agar medium.

#### 3.3.2. Agar Diffusion Medium

The synthesized compounds were tested for their antimicrobial activity using the agar diffusion method (Cruickshank et al. 1975) [38]. 

## 4. Conclusions 

Herein, in the present study, we reported the synthesis of a novel set of benzimidazole-triazole hybrid analogues, and the compounds were characterized by means of FT-IR, ^1^H-NMR, ^13^C-NMR, ESI-MS, and elemental analysis. All the compounds were evaluated for their antimicrobial activity against different strains of bacteria and fungi. Looking at the biological activity, we can conclude that all the compounds showed good to excellent results. In addition, the in vitro findings of the compounds were validated with the help of the molecular docking study with the target enzyme DNA gyrase B by investigating its binding modes of interaction. The molecular docking study revealed that all the synthesized compounds exhibited good binding energy toward the target enzyme. The in silico and in vitro findings showed that compounds **6a**,**b** were the most active against bacterial strains. The results suggested that the compounds **6a**,**b** could be used as promising scaffolds for the design and development of potential antimicrobial inhibitors. 

## Figures and Tables

**Table 1:** Energy-based interactions and intermolecular bindings between the prepared compounds and the target enzyme.

	2D Structure	Binding Energy kcal/mol	Docked Complex (Amino Acid–Ligand) Interactions	Distance (Å)
**Ciprofloxacin**	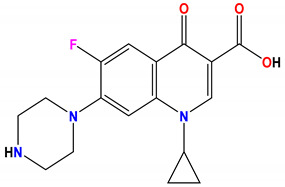	−7.4	**H-bond** Arg76:NH1—ciprofloxacin Thr165:OG1—ciprofloxacin Val43:O—ciprofloxacin	3.00 2.74 2.04
**1a**	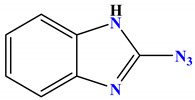	−6.4	**H-bond** Val71:O—compound **1a** **arene-σ** Asn46:CB—compound **1a**	1.87 3.91
**1b**	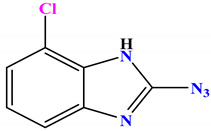	−6.5	**arene-σ** Asn46:CB—compound **1b**	3.93
**2a**	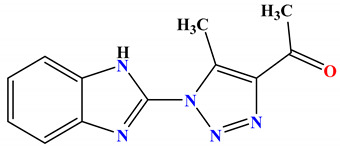	−7.9	**H-bond** Ala47:N—compound **2a** Thr165:OG1—compound **2a** Thr165:OG1—compound **2a** **arene-cation** Arg76:NH1—compound **2a**	2.99 2.98 2.95 4.06
**2b**	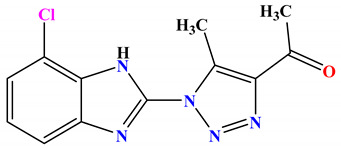	−8.1	**H-bond** Ala47:N—compound **2b** Thr165:OG1—compound **2b** Thr165:OG1—compound **2b** **arene-cation** Arg76:NH1—compound **2b**	2.99 2.95 2.94 4.02
**4a**	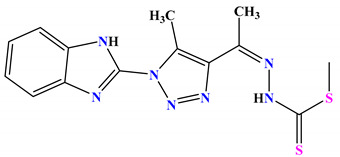	−7.0	**H-bond** Thr34:OG1—compound **4a** Thr34:OG1—compound **4a**	2.98 3.00
**4b**	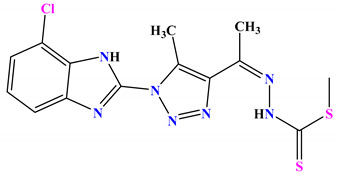	−7.1	**H-bond** Asp49:OD2—compound **4b**	2.20
**6a**	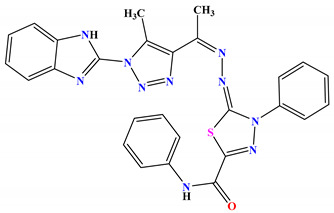	−9.7	**H-bond** Thr165:OG1—compound **6a**	2.45
**6b**	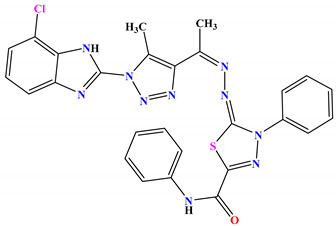	−9.8	**H-bond** Asp49:OD1—compound **6b**	2.40

**Table 2:** ADMET and drug-likeness prediction of newly prepared molecules.

	Ciprofloxacin	Compound 1a	Compound 1b	Compound 2a	Compound 2b	Compound 4a	Compound 4b	Compound 6a	Compound 6b
Molecular Weight (g/mol)	331.13	159.15	193.60	241.25	275.70	345.46	379.90	534.61	569.05
BBB permeant	No	No	Yes	No	Yes	No	No	No	No
%Human Intestinal Absorption (HIA+)	97.95	99.51	99.57	100.00	100.00	99.20	99.32	100.00	100.00
logp	−0.7	2.80	3.43	1.72	2.35	2.80	3.43	3.91	4.38
TPSA A^2^	74.57	78.44	78.44	76.47	76.47	83.79	83.79	131.05	131.05
HBA	6	5	5	6	6	7	7	11	2
HBD	2	1	1	1	1	2	2	2	6
N rotatable	3	1	1	2	2	5	5	6	1
N violations	0	0	0	0	0	0	0	1	1
Volume A^3^	285.46	133.76	147.29	209.43	222.97	288.66	302.20	454.50	468.04
Carcinogenicity	Noncarcinogenic	Noncarcinogenic	Noncarcinogenic	Noncarcinogenic	Noncarcinogenic	Noncarcinogenic	Noncarcinogenic	Noncarcinogenic	Noncarcinogenic
GI absorption	High	High	High	High	High	Low	Low	Low	Low
Bioavailability score	0.55	0.55	0.55	0.55	0.55	0.55	0.55	0.17	0.17

**Table 3:** In vitro antimicrobial screening of the newly synthesized derivatives using the agar diffusion method.

Compounds		Microorganism Inhibition Zone Diameters Using the Agar Diffusion Method (mm)			
	Gram (+ve) Bacteria	Gram (−ve) Bacteria		Fungi	
	*Staphylococcus aureus*	*Escherichia* *coli*	*Pseudomonas aeruginosa*	*Aspergillus niger*	*Candida albicans*
**2a**	15 ± 0.14	12 ± 1.08	22 ± 1.01	−ve	−ve
**2b**	−ve	5 ± 0.2	−ve	30 ± 1.16	27 ± 1.1
**4a**	23 ± 0.8	−ve	13 ± 0.65	−ve	−ve
**4b**	−ve	−ve	12 ± 08	14 ± 0.15	19 ± 1.04
**6a**	24 ± 0.6	25 ± 0.9	17 ± 0.75	20 ± 0.9	16 ± 0.89
**6b**	29 ± 1.2	21 ± 1.14	19 ± 0.79	18 ± 0.12	14 ± 0.58
Ciprofloxacin	20 ± 0.9	23 ± 1.02	21 ± 0.9	−ve	−ve
Nystatin	−ve	−ve	−ve	22 ± 0.18	23 ± 1.15

Values are expressed as mean ± standard deviation.

## Data Availability

Not applicable.

## Supplementary Materials

The following are available online. Figure S1. The molecular interactions of the other docked compounds and standard drug with the target enzyme DNA gyrase B. Left side (2D): the residues are represented in 3 letter codes. Hydrogen bonds are represented by green and blue lines and pi-interactions are represented by orange lines. Right side (3D): the docked compounds are represented by gray stick models, and the active site pockets are shown by blue stick models. H-bond interactions are shown in green dashed lines. π-interactions are shown in orange lines.

## Sample Availability

Samples of the newly synthesized compounds are available from the authors.
