# Supplementary material for: Synthesis, Identification, Computer-Aided Docking Studies, and ADMET Prediction of Novel Benzimidazo-1,2,3-triazole Based Molecules as Potential Antimicrobial Agents
Source: Molecules. 2021 Nov 25;26(23):7119. doi: 10.3390/molecules26237119 (PMC8659132; doi:10.3390/molecules26237119)
Supplement: Supplementary file 1 [file molecules-26-07119-s001.zip › molecules-1478362-supplementary.pdf]

Supplamentary file

Synthesis, Identification, Computer-Aided Docking Studies, and ADMET Prediction of Novel Benzimidazo-1,2,3-Triazole Based Molecules as Potential Antimicrobial Agents

Ciprofloxacin (Standard drug)

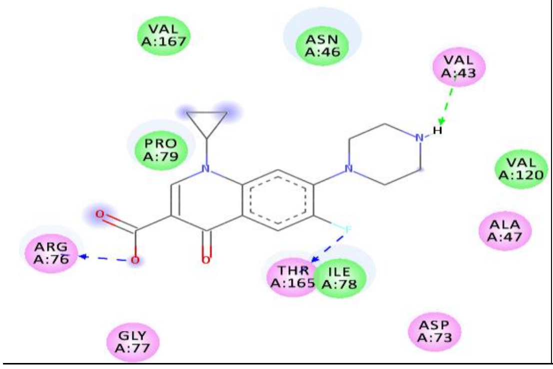

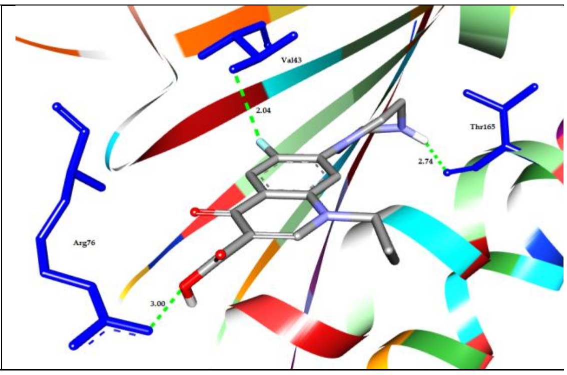

Compound 1a

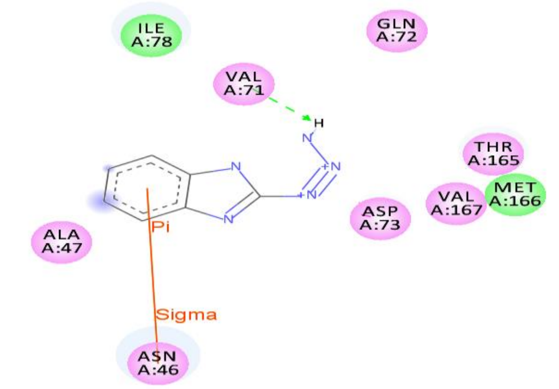

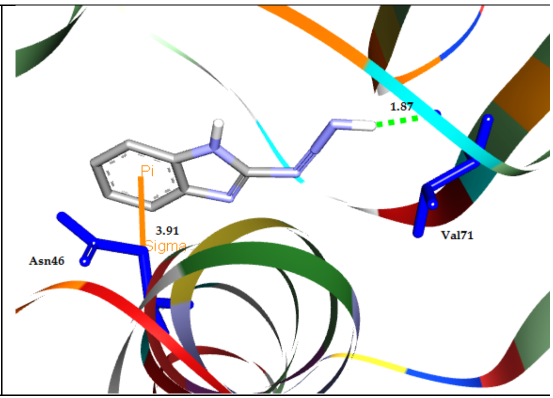

Compound 1b

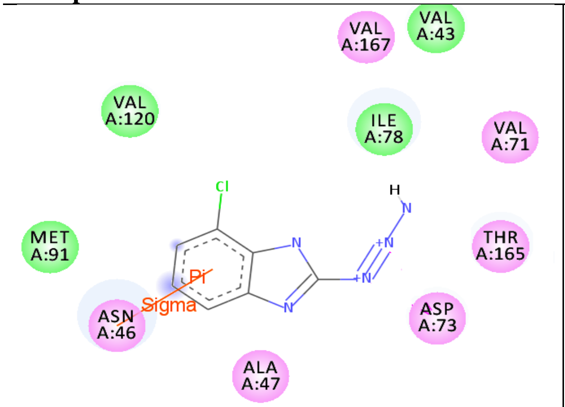

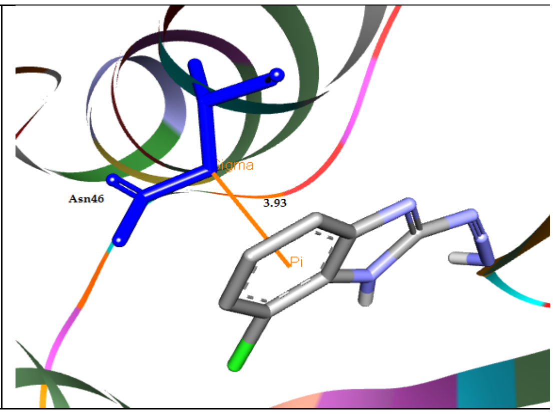

Compound 2a

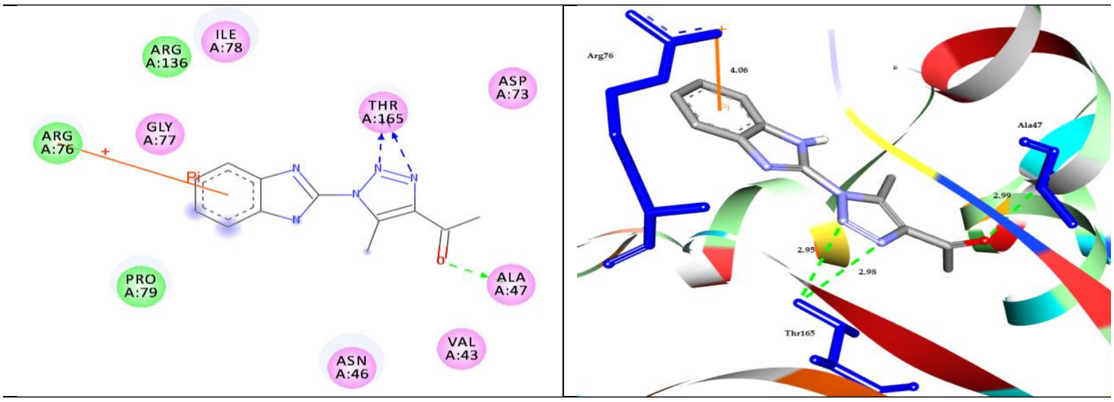

Compound 2b

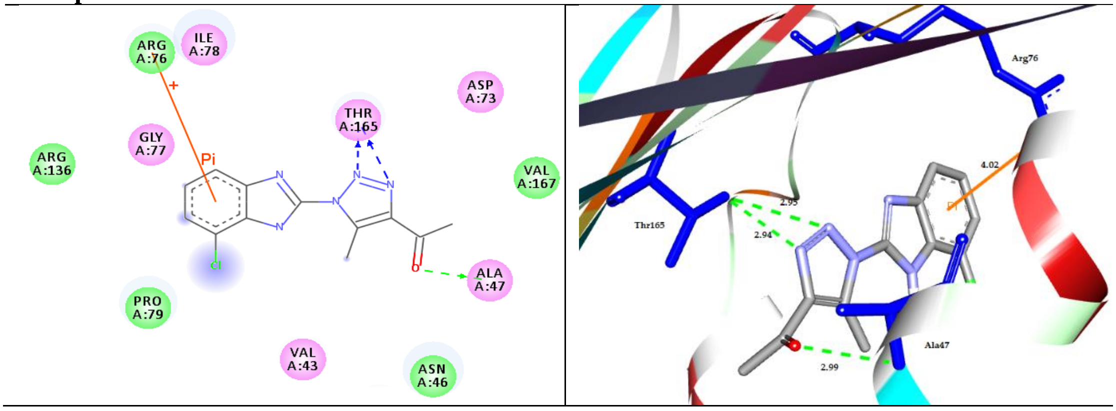

Compound 4a

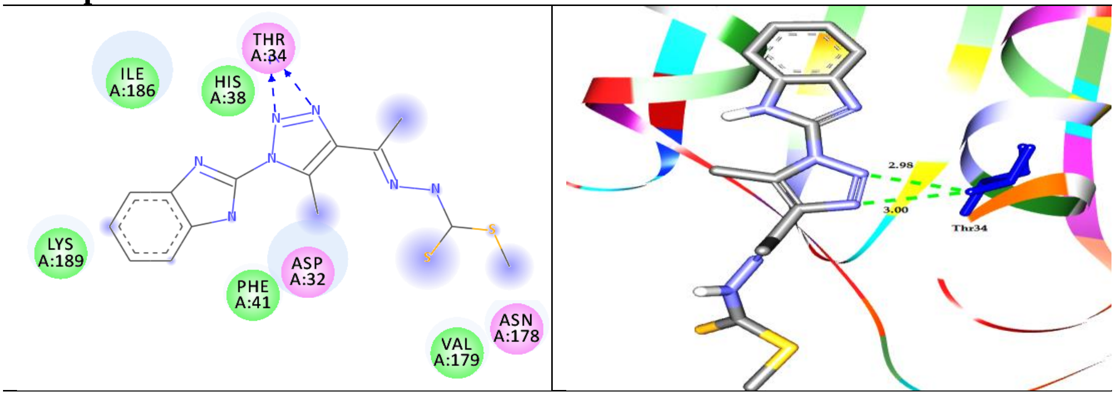

Compound 4b

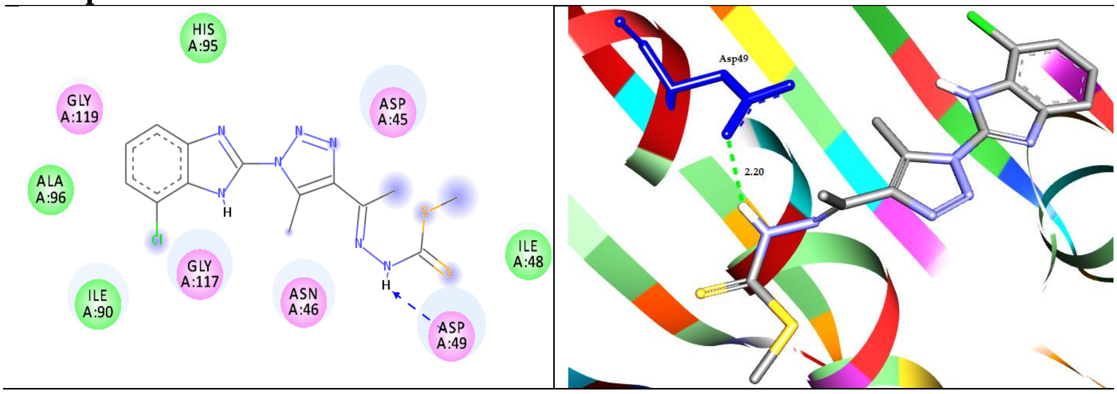

Figure S1. The molecular interactions of the other docked compounds and standard drug with the target enzyme DNA gyrase B. Left side (2D); the residues are represented in 3 letter code. Hydrogen bonds are represented in green and blue lines

and pi-interactions are represented in orange lines. Right side (3D); the docked compounds are represented in gray stick models, and the active site pockets are shown in blue stick models. H-bond interactions are shown in green dashed lines. π-interactions are shown in orange lines.
